# Microstructural models for diffusion MRI in breast cancer and surrounding stroma: an *ex vivo* study

**DOI:** 10.1002/nbm.3679

**Published:** 2016-12-21

**Authors:** Colleen Bailey, Bernard Siow, Eleftheria Panagiotaki, John H. Hipwell, Thomy Mertzanidou, Julie Owen, Patrycja Gazinska, Sarah E. Pinder, Daniel C. Alexander, David J. Hawkes

**Affiliations:** ^1^ University College LondonCentre for Medical Image ComputingLondonUK; ^2^ University College LondonCentre for Advanced Biomedical ImagingLondonUK; ^3^ King's College LondonGuy's Hospital, Breast ResearchPathologyLondonUK

**Keywords:** anisotropy, breast cancer, diffusion, DTI, *ex vivo*, MRI, restriction

## Abstract

The diffusion signal in breast tissue has primarily been modelled using apparent diffusion coefficient (ADC), intravoxel incoherent motion (IVIM) and diffusion tensor (DT) models, which may be too simplistic to describe the underlying tissue microstructure. Formalin‐fixed breast cancer samples were scanned using a wide range of gradient strengths, durations, separations and orientations. A variety of one‐ and two‐compartment models were tested to determine which best described the data. Models with restricted diffusion components and anisotropy were selected in most cancerous regions and there were no regions in which conventional ADC or DT models were selected. Maps of ADC generally related to cellularity on histology, but maps of parameters from more complex models suggest that both overall cell volume fraction and individual cell size can contribute to the diffusion signal, affecting the specificity of ADC to the tissue microstructure. The areas of coherence in diffusion anisotropy images were small, approximately 1 mm, but the orientation corresponded to stromal orientation patterns on histology.

**Abbreviations used**
ADC: apparent diffusion coefficient
AIC: Akaike information criterion
BIC: Bayesian information criterion
DT: diffusion tensor
DTI: diffusion tensor imaging
DWI: diffusion‐weighted imaging
etl: echo train length
FA: fractional anisotropy
FSE: fast spin echo
H&E: haematoxylin and eosin
IVIM: intravoxel incoherent motion
MCMC: Markov chain Monte Carlo
MRI: magnetic resonance imaging
NEX: number of averages
NST: ductal/no special type
SNR: signal‐to‐noise ratio

## 1 INTRODUCTION

Breast cancer screening allows the early detection of cancerous lesions, but improved technology increases the likelihood of detecting small, slow‐growing cancers that do not require aggressive treatment. It is estimated that 10% of women who have mammographically detected cancers would not have required treatment in their lifetime (‘overdiagnosis’),^1^, ^2^ and that post‐surgical radiation treatment may not improve 5‐year overall survival in some groups of women (a form of ‘overtreatment’).^3^

There is therefore a need for further tumour characterisation, i.e. beyond cancer detection, to identify patients in whom overdiagnosis and overtreatment are likely; magnetic resonance imaging (MRI) is particularly appealing because of its non‐invasive nature and sensitivity to microstructure. Breast cancers show great variation in microstructure: higher grades tend to have increased cell density and a more disorganised structure;^4^ immune cell infiltration and cell differentiation affect the distribution of cell types and sizes; and there are changes in the extracellular matrix related to invasion.^5^, ^6^

Diffusion MRI is sensitive to many microstructural features. Diffusion tensor imaging (DTI)^7^ and neurite orientation dispersion and density imaging,^8^ for example, have produced maps of brain architecture. In the context of cancer, methods such as VERDICT^9^, ^10^ and restriction spectrum imaging^11^ estimate tissue features such as those related to cell density. However, the exploration of breast microstructure has been relatively limited in comparison.

Clinical work has focused on data acquisition at a small number of low (≤1000 s/mm^2^) *b* values and mono‐exponential fitting to give an apparent diffusion coefficient (ADC). The ADC shows a difference between benign and malignant lesions,^12^, ^13^, ^14^, ^15^ but there is a large overlap in the ADC values of the two groups in many studies, with an area under the receiver operating curve ranging from 0.72 to 0.97 for ADC alone.^16^, ^17^ Attempts to use ADC to distinguish histological grades have produced mixed results, even with large numbers of patients.^18^, ^19^, ^20^ Some variation in ADC with molecular subtype has been observed,^20^, ^21^, ^22^ although results may be affected by the inclusion of necrotic regions common in triple‐negative cancers.

However, ADC assumes that all of the water in a particular voxel can be represented by a single ADC. In reality, intracellular water is at least partly restricted by the cell membrane, extracellular diffusion depends on the extracellular space and organisation of cells, and anisotropic structures in the tissue produce diffusion orientation dependence. The use of an inappropriate model yields ADC values that are dependent not just on physiology, but on the choice of *b* value itself,^23^ making comparisons between different scan protocols and between centres with different scanners difficult. The results from a given patient might also be difficult to interpret: although ADC correlates with cellularity in breast tumours,^24^, ^25^ immune responses and changes to the extracellular environment may also affect diffusion. More biologically motivated models have the potential to separate microstructural features related to cell proliferation from those caused by the immune response, invasion or less common cancer subtypes, and provide more specific features for tumour characterisation.

Recent clinical studies have begun to explore models beyond ADC. Intravoxel incoherent motion (IVIM) studies have looked at the vasculature in and around the tumour.^16^, ^26^, ^27^ Diffusion kurtosis results suggest that diffusion is non‐Gaussian and high‐*b*‐value measurements contain additional information.^28^, ^29^ Kurtosis can also be combined with IVIM.^30^ Diffusion tensor (DT) modelling has produced inconsistent results, generally demonstrating lower fractional anisotropy (FA) in cancers compared with normal tissues,^31^, ^32^ but not always showing a distinction from benign lesions;^15^, ^33^, ^34^ other anisotropy metrics may be more sensitive.^35^ Furthermore, the source of anisotropy is uncertain: a preclinical breast cancer model observed lower FA in hypoxic regions with lower collagen fibre content,^36^ but the differences between normoxic and hypoxic FAs were small, approximately 0.03. Other groups have suggested partial restriction in the breast ducts,^31^, ^35^ although the average duct diameter (approximately 90 μm in normal breast and larger in patients with ductal carcinoma *in situ* 37) is much larger than the average distance travelled by water molecules during typical diffusion MRI experiments.

In this article, we examined the microstructure in a small set of cancer‐containing, formalin‐fixed breast tissue samples *ex vivo*. This allowed for high spatial resolution and histopathological comparison that might shed light on the source of diffusion signal differences. For example, a previous study found large differences in the ADC of epithelial cell regions compared with surrounding stroma, as well as qualitative differences in anisotropy.^38^ The *ex vivo* approach also permitted longer scan times to obtain data over a broad range of gradient strengths, durations, orientations and diffusion times. This rich dataset was then fitted with a set of candidate models which describe the intracellular and extracellular spaces with different shapes and degrees of restriction. Model parameters were then compared with the histological features. This information can be used to optimise clinical scan protocols^39^ and to select a biologically relevant signal model with parameters that might allow for higher specificity in tumour characterisation.

## 2 METHODS

### 2.1 Samples

Seven breast tissue samples containing invasive breast cancers [two grade 1 ductal/no special type (NST), one grade 3 mucinous, four grade 3 NST] were obtained from six patients through the King's Health Partners Cancer Biobank. The use of tissue and data was approved under NHS REC agreement (07/H0874/131). Tumours ranged in size from 15 to 70 mm, with a portion at the edge of the main tumour and its surrounding stroma cut by the biobank for scanning and subsequent histology. Samples were immersed in formalin within 30 min. Before imaging, the specimens were rehydrated with phosphate‐buffered saline for at least 2 weeks and, immediately prior to imaging, samples were transferred to Fomblin Perfluorosolv PFS‐1 (Solvay Solexis, Watford, UK).

### 2.2 MRI scan procedure

Images were acquired on a 20‐cm bore, 9.4‐T MRI scanner (Varian Inc., Palo Alto, CA, USA) using a 33‐mm quadrature coil (RAPID Biomedical, Rimpar, Germany) and gradients capable of 1000 mT/m. The temperature was monitored and maintained at 18.4 ± 0.4°C.

Diffusion images were acquired with a fat‐saturated, multi‐slice, fast spin echo (FSE) sequence [resolution, 250 × 250 μm^2^; slice thickness, 500 μm; field of view, 3.2 × 3.2 cm^2^; TR = 1 s; echo train length (etl) = 4]. Forty‐two diffusion‐weighted images were acquired in three directions with a corresponding *b* = 0 image at the gradient durations, strengths and separations outlined in Table 1; higher gradient strengths used more than one average (number of averages, NEX) as indicated in parentheses. Diffusion tensor images (42 directions + six unweighted images, *δ* = 4.5 ms, *Δ* = 20 ms, TE = 30 ms) were acquired at 187 and 226 mT/m (corresponding to *b* values of 1000 and 1500 s/mm^2^, respectively). For the single case shown in Figure 2g, only a single DT image at 187 mT/m was available as a result of acquisition errors. The total diffusion scan protocol was 3 h 45 min in duration.

**Table 1:** Diffusion‐weighted imaging (DWI) scan parameters. The *b* values corresponding to the gradient strengths (*G*) at each gradient duration (*δ*) and separation (Δ)/TE. The number of averages is given in parentheses for cases in which more than one average was performed

*Δ*/TE (ms)	*δ* (ms)	*G* (mT/m)									
		40	80	120	160	200	240	280	320	360	400
10/18	3	9	37	83	148	232	334	455	594	752	928
30/45	3	30	120	269	478	748 (4)	1077 (8)				
	10	306	1222	2750	4889	7638 (4)	10999 (8)				
60/75	3	61	243	548	973 (2)	1521 (8)					
	10	649	2597	5843	10388 (2)	16231 (8)					
80/95	3	81	326	733 (2)	1303 (4)	2037 (10)					
	10	878	3514	7906 (2)	14054 (4)	21960 (10)					

The nature of the *T*_2_ decay was probed using a multi‐echo, multi‐spin sequence (TE = 5 ms; TR = 3 s; NEX = 4; 32 echoes; scan time, 26 min), and a high‐resolution (125 × 125 × 500 μm^3^) *T*_2_‐weighted image (FSE; TE = 20 ms; TR = 4 s; effective TE = 48 ms; etl = 8; NEX = 8; scan time, 17 min) was acquired to aid registration to histology. Two of the samples underwent repeat scanning either 4 days or 5 weeks apart, and were registered^40^ to examine the reproducibility.

### 2.3 MRI data analysis

The one‐ and two‐compartment models outlined in Table 2 were fitted to the data voxel‐wise, excluding voxels that were predominantly fat or with non‐mono‐exponential *T*_2_ decay (non‐mono‐exponentiality was defined as a main peak area comprising less than 90% of the total spectral area in the *T*_2_ spectrum from non‐negative least‐squares analysis^41^). Compartment shapes are described in detail in Panagiotaki et al.^42^ and are summarised in the Appendix: a Ball describes unrestricted (free or hindered) isotropic diffusion; a Tensor describes anisotropic free diffusion (with diffusion coefficients *D*_1_, *D*_2_ and *D*_3_ in three orthogonal directions characterised by angles *θ* and *ϕ* for the primary diffusion direction and *α* describing the angle of the secondary diffusion direction in the perpendicular plane); a Zeppelin is a cylindrically symmetrical tensor; and a Sphere describes diffusion restricted isotropically by an impermeable membrane with radius *R*. In this nomenclature, the conventional ADC model is represented by a Ball and a bi‐exponential fit by Ball–Ball. In addition to the diffusion, shape and orientation parameters for each compartment shown in parentheses in Table 2, two‐compartment models have extracellular and intracellular volume fractions *f*_E_ and *f*_I_, respectively, and all models include the equilibrium signal *S*_0_ and the *T*_2_ relaxation time constant as fitting parameters.

**Table 2:** Models tested (with fitting parameters in parentheses). Compartment shapes are described in the text and Appendix

Models tested	Extracellular compartment	Intracellular compartment	No. of fitting parameters
Ball (ADC)	Ball (*D*_1_)		3
Zeppelin	Zeppelin (*D*_1_, *D*_2_, *θ*, *ϕ*)		6
Tensor (DT)	Tensor (*D*_1_, *D*_2_, *D*_3_, *θ*, *ϕ*, *α*)		8
Ball–Ball	Ball (*D*_1_)	Ball (*D*_I_)	5
Zeppelin–Ball	Zeppelin (*D*_1_, *D*_2_, *θ*, *ϕ*)	Ball (*D*_I_)	8
Tensor–Ball	Tensor (*D*_1_, *D*_2_, *D*_3_, *θ*, *ϕ*, *α*)	Ball (*D*_I_)	10
Ball–Sphere	Ball (*D*_1_)	Sphere (*D*_I_, *R*)	6
Zeppelin–Sphere	Zeppelin (*D*_1_, *D*_2_, *θ*, *ϕ*)	Sphere (*D*_I_, *R*)	9
Tensor–Sphere	Tensor (*D*_1_, *D*_2_, *D*_3_, *θ*, *ϕ*, *α*)	Sphere (*D*_I_, *R*)	11

Data were fitted using an iterative maximum likelihood procedure that accounts for local minima and Rician noise.^42^ The noise was derived from correction of the standard deviation of signal in an empty region^43^ in each image. Parameters were constrained as follows: 0.01 < *D* < 3 mm^2^/s for all diffusion coefficients; *f*_E_ + *f*_I_ = 1; 0.1 < *R* < 20 μm; 0 < *S*_0_; 0.001 < *T*_2_ < 3 s. Model selection was performed using the Akaike information criterion (AIC), AIC =  − 2 ln *L* + 2*k*,^44^ and the Bayesian information criterion (BIC), BIC =  − 2 ln *L* + *k* ln *n*,^45^ where *L* is the maximum likelihood of the model given the data, *k* is the number of fitting parameters and *n* is the number of data points.

Parameter distributions were examined using a Markov chain Monte Carlo (MCMC) procedure^39^ for selected voxel data. Using a Metropolis–Hastings sampler, 500 samples were drawn from the posterior distribution with the optimised parameter values as a starting point, a burn‐in of 5000 iterations, a sampling interval of 400 and a Gaussian proposal distribution with standard deviation equal to 1% of the initial parameter estimate.

### 2.4 Histology and registration

After imaging, the samples were processed and embedded in paraffin wax, and sections approximately 3 μm thick were cut at every 100 μm through the block. The slides were stained with haematoxylin and eosin (H&E), and digitised using a C9600–01 NanoZoomer Digital Slide Scanner (Hamamatsu, Hamamatsu City, Japan) at 20× magnification (21 708 pixels/cm).

Histological images were stacked into a volume using two‐dimensional pairwise registrations between adjacent slices based on a block‐matching strategy.^46^, ^47^ The transformation model used was rigid body and the similarity measure was the correlation coefficient. This pairwise registration aligns each slice with the subsequent slice and then concatenates transformations to generate a volume consisting of stacked slices registered with respect to a reference slice in the middle of the image stack. The slice separation was taken as 100 μm.

To register the stacked histology volume to the *T*_2_‐weighted MRI, 9–15 manually selected corresponding landmarks were identified in each MRI/histology volume pair. This provided an approximate initial alignment as a result of the differing volume orientations. For the final registration, the volumes were resampled to isotropic voxels (0.5 mm for MRI and 0.1 mm for histology) to reduce orientational bias and the relevant regions (bright foreground voxels in MRI and non‐zero voxels in histology) were selected to restrict the region over which the similarity measure was calculated to the internal tissue contrast. An intensity‐based affine registration from ITK^48^ was then performed using normalised mutual information as the similarity measure and a regular step, single‐scale optimisation with MRI as the target volume. The shear component of the affine registration will, to first order, correct for any residual cumulative stacking error of the histology slices. A sample of the three‐dimensional histology stack in each of three orthogonal views is shown in Figure S1 with the diffusion MRI slice overlaid. As a result of the orientation of the MRI slice with respect to the histological slicing plane, only a portion of the histology slice corresponds to the diffusion image, which is indicated by outlines of tumour regions in subsequent figures. The transformation was also applied to the primary diffusion vectors to keep alignment consistent with the image orientation.

## 3 RESULTS

Fitting quality, parameter reproducibility and posterior parameter distributions were first examined as part of the model selection process. In a second section, model parameters were compared with histology, first examining parameters associated with compartment size and restriction, and then those associated with orientational structure.

### 3.1 Fitting results and model selection

**Figure 1:**
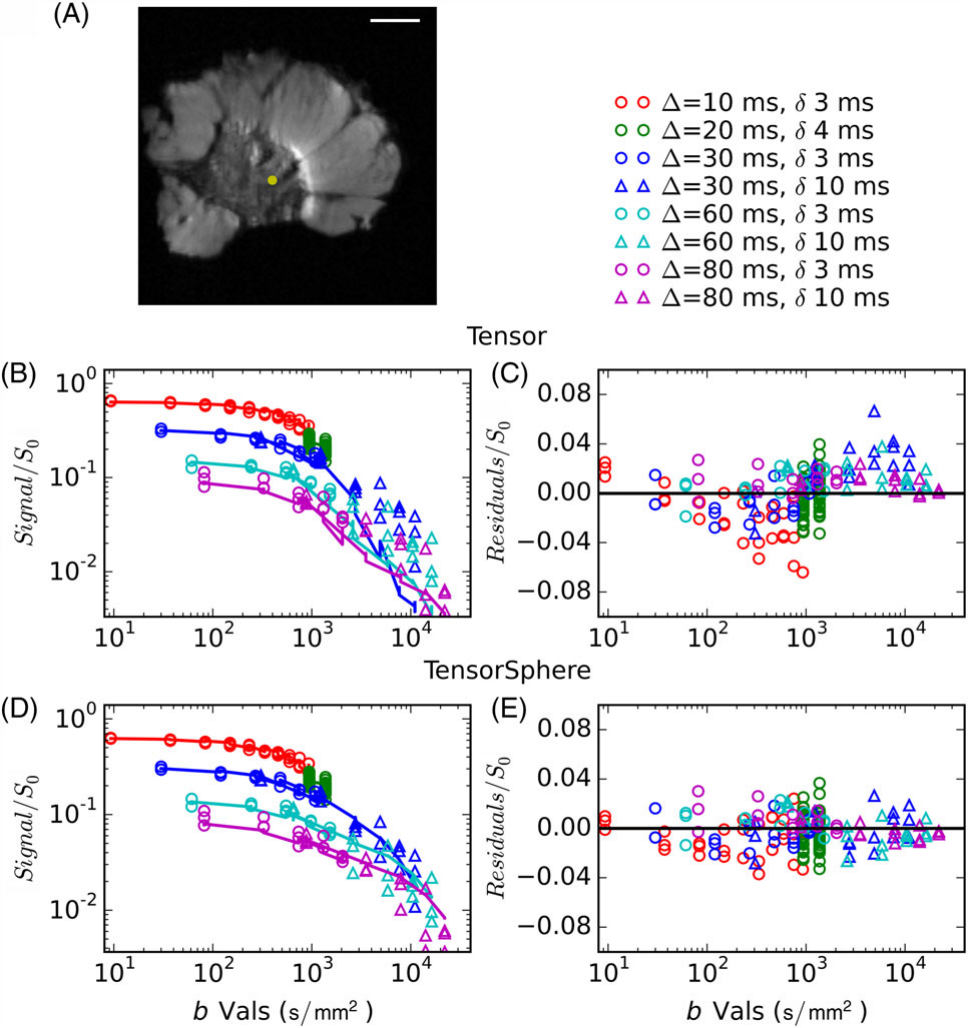
(a) Sample diffusion‐weighted image (DWI) (*b* = 1076 **s/mm** ^**2**^) with the voxel of interest marked in yellow. (b, d) DWI (each gradient separation *Δ* is a different colour; all directions for a particular *Δ* are plotted with a single colour) and DTI (green) data (points) as a function of *b* value with full lines showing the fit for the Tensor model (b) and the Tensor–Sphere model (d). Corresponding residuals (c and e) show systematic errors for the Tensor model. White scale bars in all magnetic resonance images represent 5 mm

Figure 1a shows a sample diffusion‐weighted image (*b* = 1076 s/mm^2^, *Δ* = 30 ms, *δ* = 3 ms); the yellow point indicates the voxel for which the data (points) are plotted in Figure 1b–d. Fits (full lines) are shown for the standard DT model (b) and the Tensor‐Sphere model (d) (fits for other models can be seen in Figure S2). The plotted fitted lines are calculated assuming Rician noise (mean noise/*S*_0_ = 0.01 for this voxel), so that any remaining systematic bias should be the result of model choice. Values are normalised using the fitted *S*_0_ value. The residuals (Figure 1c, e) emphasise that even the most complex single‐compartment model, the Tensor, overestimates the signal at low diffusion times and *b* values, and underestimates the signal at high diffusion times and *b* values. This voxel is typical of those that have higher fitted *f*_I_ values in two‐compartment models (*f*_I_ = 0.44 for the Tensor–Sphere in this voxel); fits for voxels with lower *f*_I_ deviate less from the data, but have a stronger orientational dependence.

**Figure 2:**
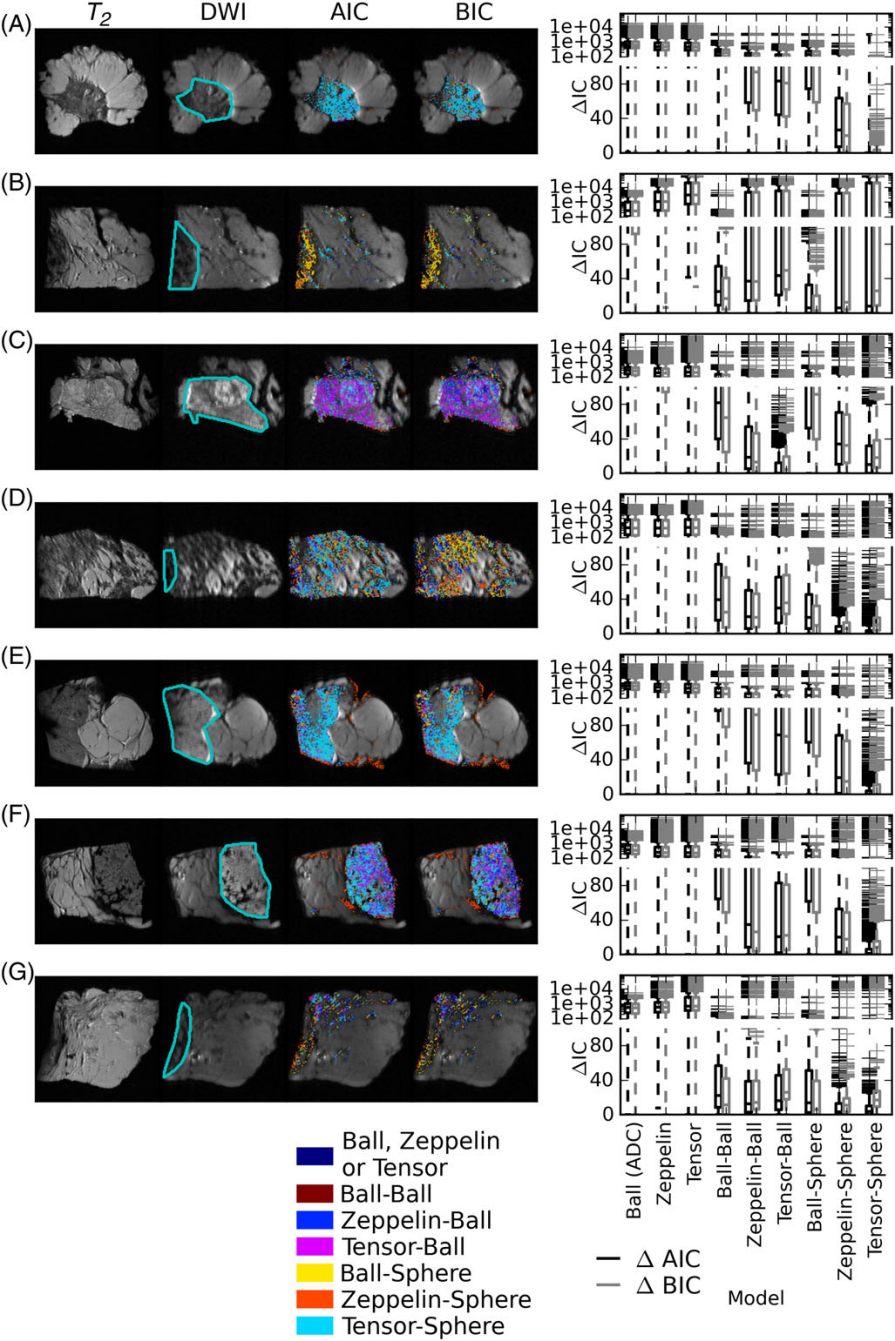
*T*_2_‐weighted images (first column) and diffusion‐weighted images (second column) for each sample with the main tumour focus outlined in cyan. Maps demonstrate the model that best explains the data in each voxel [third column, Akaike information criterion (AIC); fourth column, Bayesian information criterion (BIC)] and the distribution of relative AICs and BICs (low values indicate better explanation) across the samples. (a, b) Grade 1 ductal/no special type (NST); (c) grade 3 mucinous; (d–g) grade 3 NST. Diffusion data from most voxels are best explained by an anisotropic compartment and a restricted compartment (Zeppelin–Sphere or Tensor–Sphere), although there are several regions, particularly in the grade 3 mucinous carcinoma, in which no restriction is required to explain the data (Zeppelin–Ball, Tensor–Ball). Three samples (b, d and g) contained more fat, with only small tumour areas at the edge of the sample and some voxels exhibiting isotropic restriction (Ball–Sphere)

*T*_2_‐weighted and diffusion‐weighted images for each sample are shown in the two left‐most columns of Figure 2 with the tumour focus outlined in cyan. Most of the remaining tissue is fat; some fibroglandular voxels outside the main tumour focus are also present. The best model for each voxel in each sample is shown in the third column of Figure 2 for AIC and in the fourth column for BIC (regions with no colour were excluded either as fat or for having non‐mono‐exponential *T*_2_). The distributions of relative AICs and BICs across each sample are shown in the boxplots, with the line at the median, the box extending to the quartile values and the whiskers showing the range. The four larger samples (Figure 2a, c, e and f) are best explained by models with anisotropy; the remaining three cases (Figure 2b, d and g) include large amounts of fat, with a smaller tumour focus near the edge of the field of view, where the Ball–Sphere model is selected in some voxels. This could be a result of signal‐to‐noise ratio (SNR) issues at the image edge, contamination from interspersed fat, or may be a true biological difference (the case with the largest number of voxels best explained by the Ball–Sphere model is an invasive carcinoma of histological grade 1 and NST). Many voxels are best explained by a model with a restricted Sphere component, but some regions, particularly in the grade 3 mucinous carcinoma (Figure 2c), are better explained by an unrestricted Zeppelin–Ball or Tensor–Ball model. There are no voxels in any of the samples in which a conventional ADC or DT is the best choice. Subsequent results focus on the four samples with large central sections of invasive cancer (Figure 2a, c, e, f), but the remaining cases can be seen in the Supporting Information.

**Figure 3:**
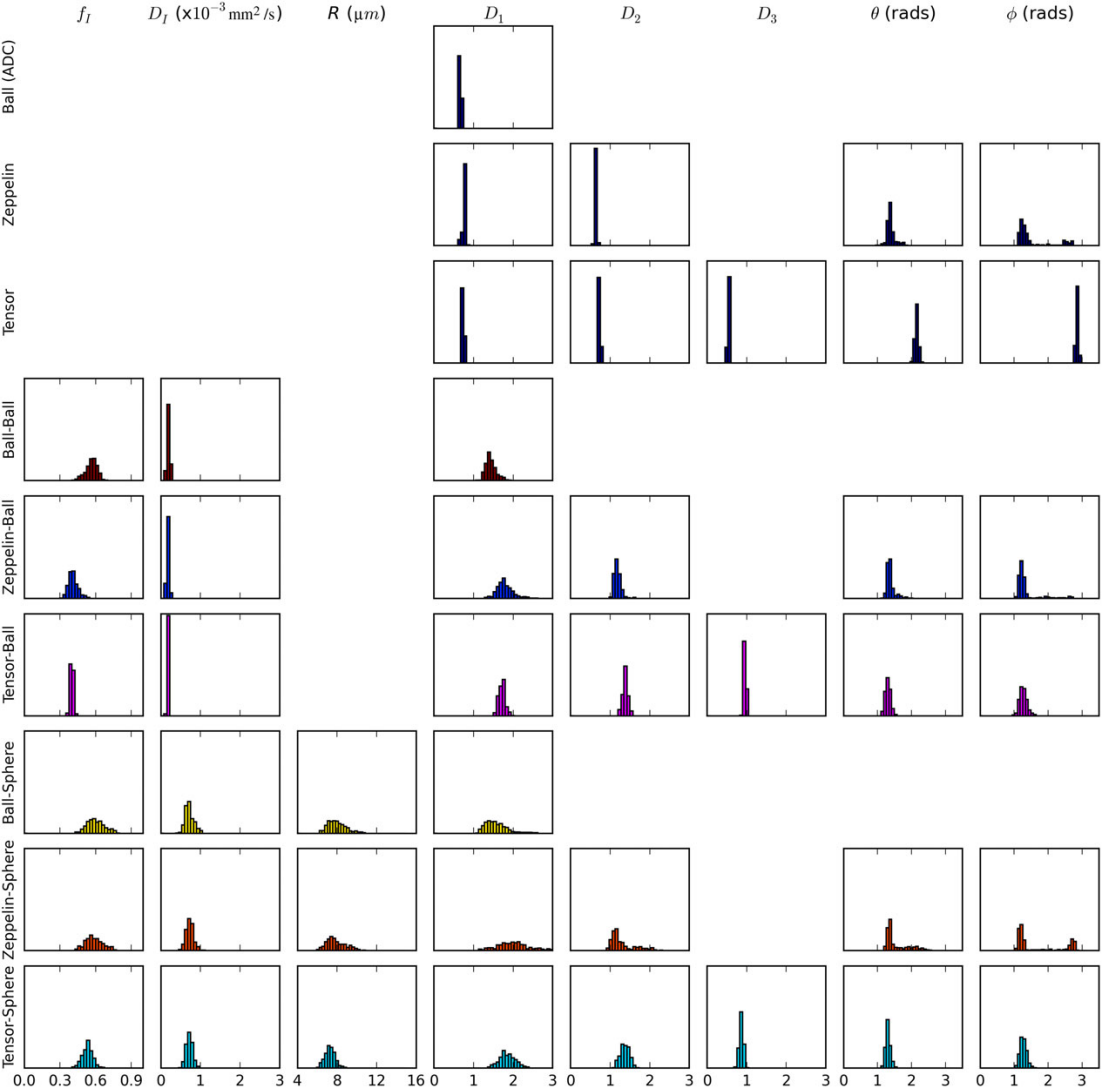
Histograms of the posterior parameter distributions for each model obtained using the Markov chain Monte Carlo (MCMC) procedure with data for a single voxel. Mean values and parameter distributions are similar for all restricted models (with Sphere compartment), aside from the extracellular diffusion coefficients, *D*_1–3_, demonstrating that increasing model complexity does not affect parameter stability substantially

Figure 3 demonstrates the parameter variance using histograms from the MCMC procedure for data from a single voxel with moderately high *f*_I_. The width of the diffusion coefficient distributions is larger for the two‐compartment models, but the distributions of the angular parameters are similar. The mean values are similar across two‐compartment models with restriction (Ball–Sphere, Zeppelin–Sphere, Tensor–Sphere) for *f*_I_, *D*_I_, *R*, *θ* and *ϕ*, but differ for related diffusion coefficients (e.g. *D*_1_ from Ball–Sphere is between *D*_1_ and *D*_2_ for Zeppelin–Sphere). Histograms from voxels with lower *f*_I_ (see Figure S3) showed similar patterns, but with narrower *θ* and *ϕ* distributions, probably because of the larger extracellular signal.

There was generally good agreement between the posterior distributions and parameter maps (see Figure S4) for the Zeppelin–Sphere and Tensor–Sphere models. Reproducibility (Figure S5) was also similar, and so subsequent results are presented for the simpler Zeppelin–Sphere model.

### 3.2 Model parameters – *f*_I_ and *R*

**Figure 4:**
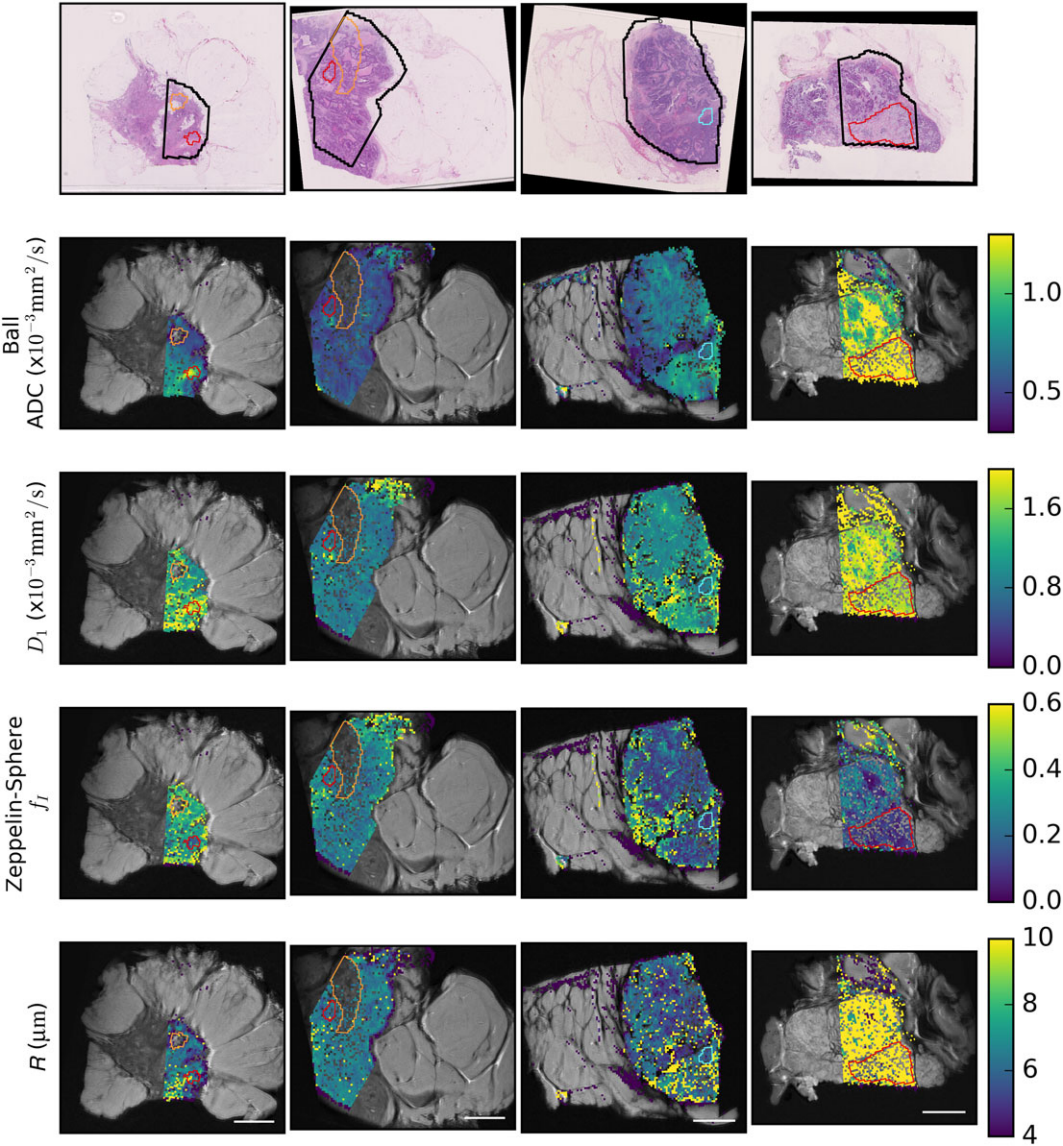
Parametric maps for the apparent diffusion coefficient (ADC, second row) and selected parameters from the Zeppelin–Sphere model. Regions of low cellularity and correspondingly high ADC are outlined in red and typically correspond to lower *f*_I_ in the Zeppelin–Sphere model. However, some regions (e.g. cyan outline) have high cellularity relative to their surroundings, but higher ADC, which may be explained by a larger cell radius, *R*. Additional samples are shown in Figure S6. Regions without colour were excluded either as fat (orange outline in first column) or as non‐mono‐exponential *T*_2_ (orange outline in second column), which often corresponded to necrotic regions on histology

Figure 4 shows H&E‐stained histology in the top row, parametric maps from ADC (second row) and selected parameters from the Zeppelin–Sphere model (rows 3–5). There was variation in all parameters across samples, including the characteristic high ADC in the mucinous carcinoma in the last column (mean ± standard deviation across fitted tumour voxels (×10^−^^3^ mm^2^/s): 1.3 ± 0.3 for grade 3 mucinous; 0.67 ± 0.18 and 0.50 ± 0.17 for grade 1 NST; 0.9 ± 0.6, 0.55 ± 0.09, 0.68 ± 0.14 and 0.48 ± 0.22 for grade 3 NST).

**Figure 5:**
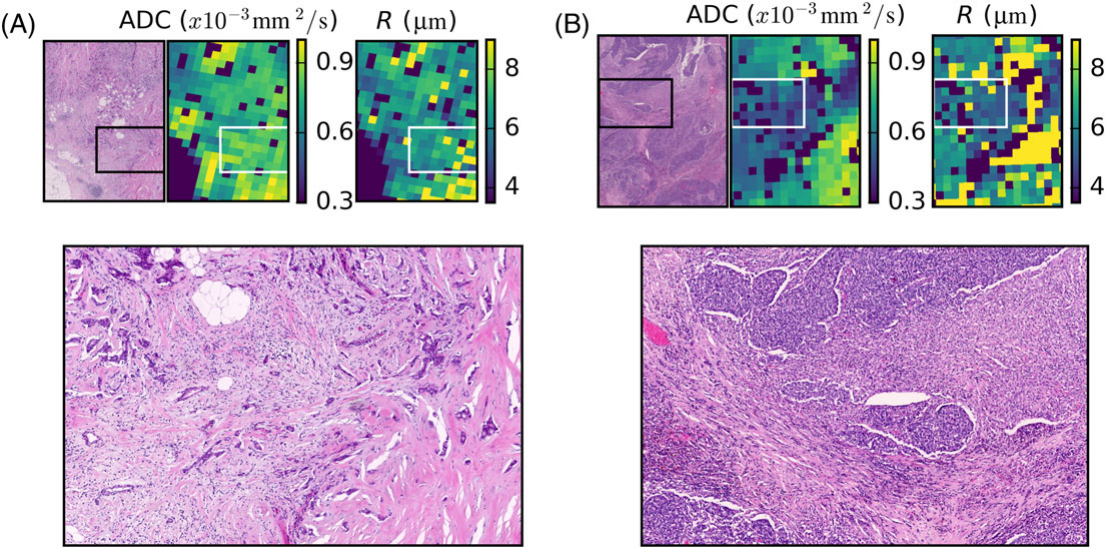
Regions from two of the tumours demonstrating different spatial variation in the apparent diffusion coefficient (ADC) and Zeppelin–Sphere model parameters. (a) The black and white boxes show regions of relatively uniform ADC in which the radius *R* from the Zeppelin–Sphere model suggests smaller cells in the left half of the region, which is supported by the histology, which shows smaller inflammatory cells on the left of the region and an increasing proportion of clusters of larger epithelial cells on the right. (b) Boxes enclose a region in which ADC is lower in the bottom right, but cellularity appears lower on histology. The high magnification histology demonstrates that the cells are smaller in this region, which is consistent with the lower values in this region of the *R* map

Regions of low cellularity on histology tend to correspond to regions of high ADC and low *f*_I_ (red outlines). However, there are regions in which higher cellularity does not correspond to lower ADC (compare the region outlined in cyan with surrounding regions) and, in such cases, the patterns in the Zeppelin–Sphere parameter maps differ from those of the conventional ADC maps. The high magnification histology in Figure 5a (from black inset on histology and corresponding to white inset on MRI parameter maps) shows a region with varying cell size as a result of the presence of immune cells amongst cancerous epithelial cells. The ADC in this region is relatively uniform [(0.77 ± 0.16) × 10^−^^3^ mm^2^/s], but *R* increases from 6.4 ± 0.4 μm on the left side of the image to 8.2 ± 3.1 μm on the right side of the image, where the proportion of larger epithelial cells increases. The boxes in Figure 5b show a region of low cellularity (near the cyan outline from Figure 4) where ADC is lower than in the surroundings [(0.51 ± 0.07) × 10^−^^3^ mm^2^/s *versus* (0.68 ± 0.08) × 10^−^^3^ mm^2^/s in the same‐sized region above], contrary to conventional thinking about ADC, but the *R* map and high magnification histology suggest that small cells in this region restrict diffusion, limiting diffusion decay in spite of the lower cellular volume fraction.

For the mucinous carcinoma (last column), the fitted *R* parameter hits the 20 μm maximum allowed by the fitting procedure in most voxels. This large *R* value is equivalent to unrestricted diffusion given the diffusion lengths probed in this experiment; thus, this finding is consistent with Figure 2 data that the Zeppelin–Ball model is a better choice in this sample.

### 3.3 Model parameters – orientation

**Figure 6:**
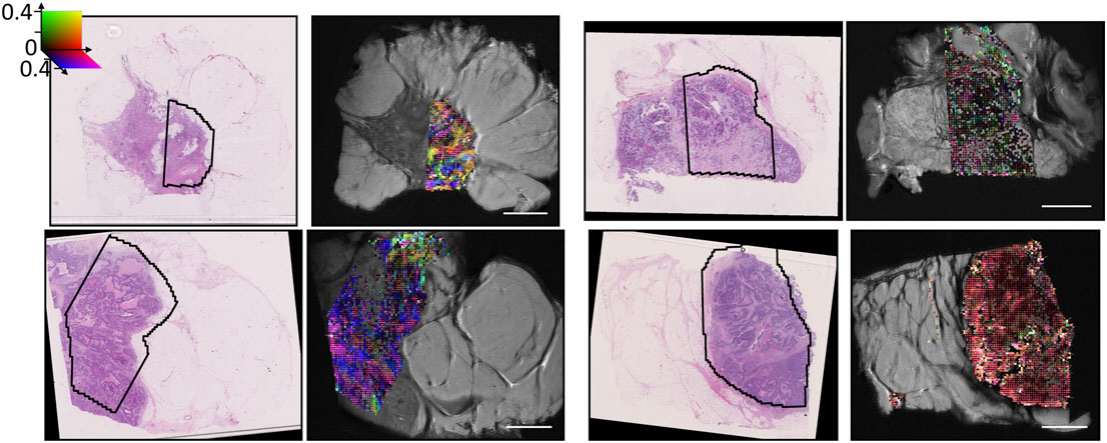
Haematoxylin and eosin (H&E)‐stained histology alongside colour fractional anisotropy (FA) maps from the Zeppelin portion of the Zeppelin–Sphere fit. Remaining samples are shown in Figure S7

**Figure 7:**
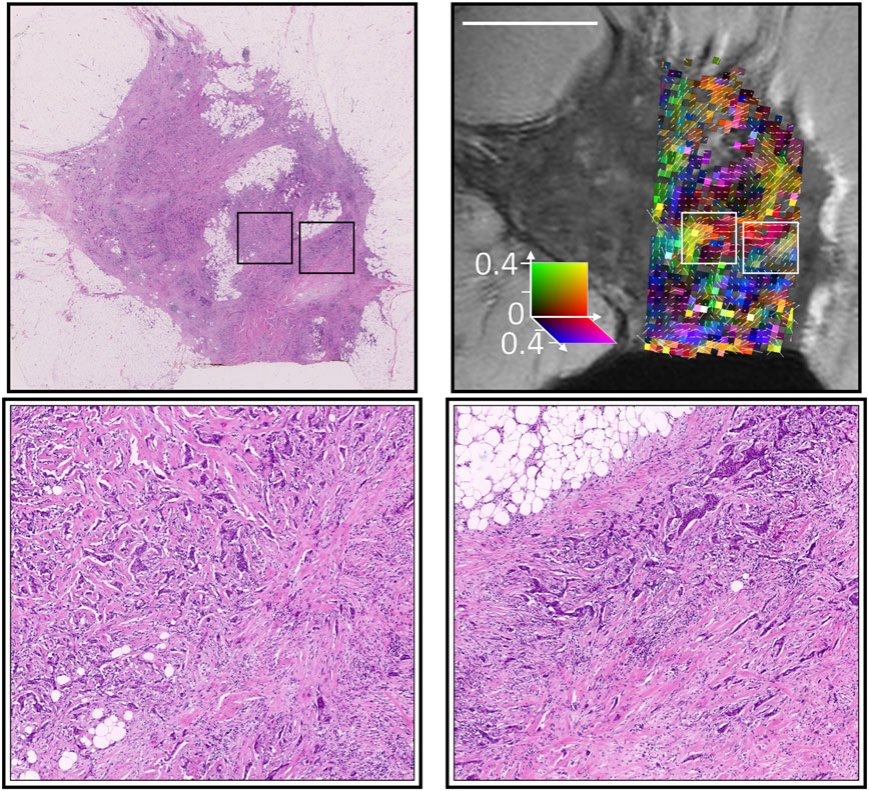
Higher magnification histology of two regions (outlined in boxes) with higher fractional anisotropy (FA), demonstrating correspondence between the primary diffusion direction and directional patterns of the fibrous stroma

**Figure 8:**
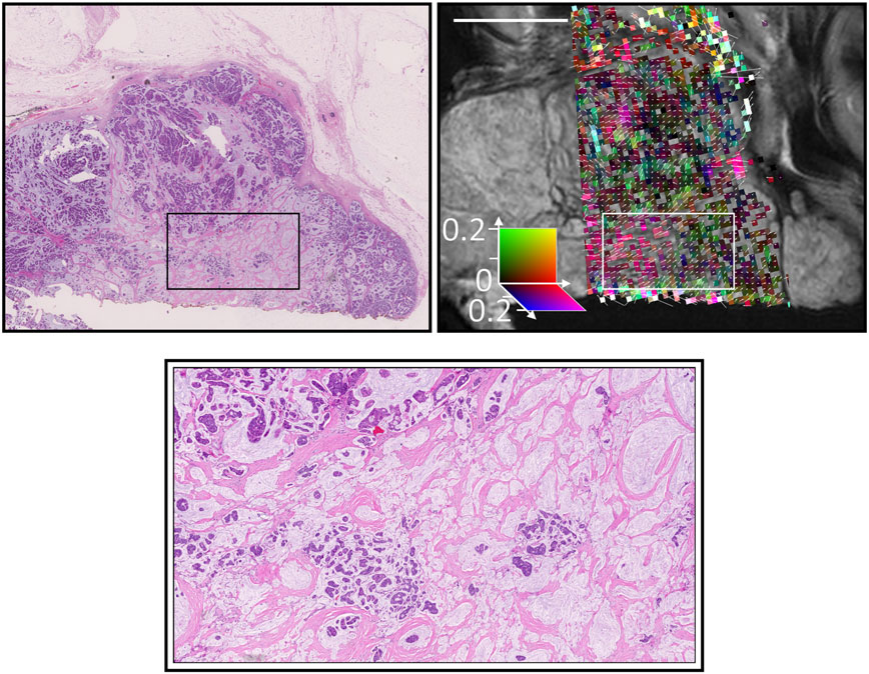
Higher magnification of the grade 3 mucinous carcinoma showing correspondence between the primary diffusion direction and directional patterns in the fibrous stroma, even in the areas of low cellularity. Note that the colour scale and arrow length have been adjusted from previous figures to better identify regions of coherence

Colour FA maps from the Zeppelin portion of the Zeppelin–Sphere model (i.e. removing the isotropic spherical component from the FA calculation) are shown alongside the H&E‐stained histology in Figure 6. Small regions of coherent direction (approximately 4 voxels =1 mm) were observed and are highlighted for regions from two samples in Figures 7 and 8.

**Figure 9:**
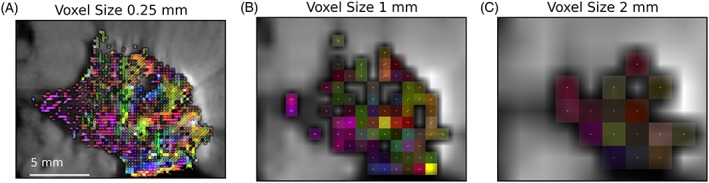
The colour fractional anisotropy (FA) map for the Zeppelin portion of the Zeppelin–Sphere model for the original 0.25 × 0.25 × 0.**5 mm** ^**3**^ data (a) downsampled (by averaging) to 1.0 × 1.0 × 0.**5 mm** ^**3**^ (b) and 2.0 × 2.0 × 0.**5 mm** ^**3**^ (c). At lower resolutions, the anisotropy becomes weaker (colours are less bright) and it is more difficult to discern a coherent direction in the data

Figure 9 displays the colour FA maps for the original data (a) and data downsampled in‐plane (b, c), demonstrating that anisotropy becomes weaker (colours less bright) at lower resolution, particularly at 2 mm resolution.

## 4 DISCUSSION

### 4.1 Model selection

This article presents detailed diffusion data of breast tissue samples acquired using a rich imaging protocol, testing a variety of one‐ and two‐compartment models with different shapes, with and without restriction. The model that best explains the data varies in different tumours and regions, which is not unexpected given the diversity of breast cancer microstructure. This variation has also been reported in *ex vivo* prostate studies.^49^ A small fraction of voxels were excluded from fitting as a result of non‐mono‐exponential *T*_2_ decay, and histology revealed that many such voxels were in necrotic regions. In most voxels, the data were best explained by the anisotropic two‐compartment models: Zeppelin–Sphere and Tensor–Sphere in regions of higher cellularity, indicating that a restricted diffusion component is present in these areas, or Zeppelin–Ball and Tensor–Ball in regions of low cellularity. There was no clear trend in parameters with grade, although the number of samples is small and the samples demonstrated heterogeneity. There were no regions in which conventional ADC or DTI best explained the data. These models are by far the most commonly used clinically, albeit with more limited single‐shell protocols consisting of lower *b* values.

Although the constraints of clinical scans limit the diffusion data that can be obtained, the results of the rich protocol suggest that there is valuable information that is not captured by most clinical protocols and diffusion models. For example, a clinical protocol with scan parameters producing signal sensitive to the observed *R* range of 6–9 μm could be designed. This approach has been successfully applied in prostate cancer to distinguish tumour from benign regions,^9^ and may improve tumour characterisation in breast.

### 4.2 Parameter values

Parameter values varied both across and within samples. The MCMC parameter distributions suggested that the fitted parameters were relatively stable, and comparison with histology further supported the hypothesis that parameter variations reflected true microstructural differences. This heterogeneity makes a simple summary of parameter values challenging. The mucinous carcinoma examined had the highest ADC (Figure 4, third column), as has been reported previously, and is attributed to low cellularity.^13^, ^15^

In other samples, the ADC in regions of low cellularity was approximately 1.3 × 10^−^^3^ mm^2^/s, which is in agreement with the value of 1.23 × 10^−^^3^ mm^2^/s for interlobular stroma found by Norddin et al.^38^ at 22°C. Regions of higher cellularity tended towards lower ADC (approximately 0.6 × 10^−^^3^ mm^2^/s), in agreement with the mean diffusivity in breast lobules (0.59 × 10^−^^3^ mm^2^/s) and regions of invasive ductal carcinoma (0.45 × 10^−^^3^ mm^2^/s), despite differences in scan parameters and resolution.^38^ However, ADC does not fully characterise the histological features: some regions with little variation in ADC (box in Figure 5a) showed variations in cellularity and cell size that more closely reflected variations in the *f*_I_ and *R* maps from the Zeppelin–Sphere model; other regions with low ADC actually had low cellularity relative to their surroundings (Figure 5b). The map of *R* suggested that this was a result of smaller cell size, and a qualitative estimate of cell size based on nuclear size in H&E supported this.

This is the first study using two‐compartment restricted diffusion models to examine *ex vivo* breast tissue. However, Lasič et al.^50^ examined MCF‐7 cells that had been grown *in vitro* and found a median size of 13.2 μm assuming a log‐normal distribution and a width of 0.6 μm. This is larger than the radius range observed in most sample regions in the present study (6–9 μm). This may reflect true biological differences – the *ex vivo* samples include stromal regions containing smaller cells such as lymphoid cells – or may indicate cell shrinkage as a result of fixation or the use of a single cell radius parameter rather than a cell size distribution. Models incorporating cell size distributions were beyond the scope of this study, but should be examined in the future.

The diffusion coefficients themselves are affected by the room temperature scan, e.g. they are lower than the intracellular (1.5 × 10^−^^3^ mm^2^/s) and extracellular (2.8 × 10^−^^3^ mm^2^/s) values observed for *in vitro* breast cell samples at 37°C.^50^ Fixation may also affect diffusion through cross‐linking and decreased water content, but work in prostate suggests that the relative signal fractions in compartments are similar before and after fixation and that changes are unlikely to affect model ranking.^51^, ^52^ Studies in brain^53^ and optic nerve^54^ also demonstrate that the microstructure and anisotropy are largely unaffected by fixation. Thus, cell size and organisational information should reflect the *in vivo* situation reasonably well, but further experiments are needed to verify this.

### 4.3 Anisotropy

Previous work^31^ has suggested that anisotropy in breast DTI might be a result of breast ductal structures. We were unable to examine this hypothesis in this study because of the limited number of normal duct structures in the samples, but anisotropy was observed in regions in which no breast ducts were present on histology. The regions with strongest anisotropy correspond to regions of lower cellularity in which H&E staining demonstrates a coherent collagen pattern. The possibility that structures not visible on H&E staining may contribute to diffusion anisotropy cannot be eliminated, but work in gels and tumour xenografts suggests that collagen results in the anisotropic diffusion of large molecules^55^ and may affect the smaller water molecules that provide the signals measured here. These findings are consistent with the higher FA observed in fibrous stroma relative to breast lobules,^38^ and with the higher FA in regions of hypoxia with increased collagen fibre density.^36^

The regions of coherence on the FA maps (Figure 6) are relatively small, approximately 1 mm, and are likely to be averaged out at resolutions approaching 2 mm (Figure 9), which may account for the inconsistency in previous clinical findings: in healthy breast tissue, the ducts and/or surrounding structures may produce large regions of anterior–posterior anisotropy;^35^ tumours disrupt this structure and lower the large‐scale anisotropy, but smaller regions of coherence with varying direction exist in the stroma, and may produce observable anisotropy depending on the image resolution, SNR and how disruptive the tumour is. For example, higher order diffusion tensor methods have been successfully applied in patients, and demonstrate the presence of multiple diffusion fibre directions in some voxels of malignant tumours, but anisotropy is not present in benign tumours.^17^ There is also evidence that collagen reorients in invasive tumours,^6^ and is more strongly aligned in malignant samples relative to hyperplastic samples.^56^ Of particular note in this study is that, although the mucinous carcinoma had lower anisotropy, small regions of coherence were still observable and corresponded to stromal orientation patterns, whereas mucinous carcinomas have proven difficult to distinguish from normal and benign tissue using conventional ADC methods.^13^ Thus, the ability to detect small regions of anisotropy within and around tumours is a potentially valuable biomarker, and may become achievable in the near future with the use of reduced field‐of‐view sequences, double diffusion encoding sequences^57^ or higher order diffusion tensor methods.^17^

### 4.4 Limitations

In addition to the use of a single average cell size parameter, and the fixation and temperature issues already discussed, this study has several limitations. The number of samples was small, but variation in the preferred microstructural model and parameters was observed even within this range of grades and histological subtypes of breast cancer. Samples were examined voxel‐wise to maximise the information obtained about different microstructural environments, but additional samples are needed to determine whether the findings are generalisable across all breast cancers.

The gradient strengths used were larger than those commonly available clinically, but Figure 1 demonstrates that single‐compartment models, such as DTI, diverge from the data even at low *b* values (e.g. red circles). More limited gradient strengths and diffusion times may result in more uncertainty in model parameters, but a priori information, such as that obtained from *ex vivo* studies and validated *in vivo*, may be useful in constraining models applied to more limited clinical data.

All models assumed no exchange of water between compartments during the measurement, although there may be some additional signal decay, particularly at long diffusion times and high *b* values, arising from exchange effects. *T*_2_ was assumed to be mono‐exponential, and a separate sequence ascertained where this assumption failed and excluded these voxels from fitting. The method could potentially be extended to include regions with multi‐exponential *T*_2_, given sufficient data. We assumed spherical cells of uniform size, which is a simplification of the real biological system. In cases in which there is some eccentricity in the cell shape, the radius estimate will represent a volume average of this parameter. Future work could extend the model selection to include compartments with anisotropic restriction; however, fitting both a cell size and compartment eccentricity using a basic pulsed gradient spin echo sequence biases both the radius and eccentricity parameters.^58^

## 5 CONCLUSIONS

This is the first study to examine such a broad range of diffusion data in human breast tissue samples and to model the data using both anisotropy and restriction. The data from most cellular cancer regions and the adjacent fibroglandular tissue were best explained using a Tensor–Sphere or Zeppelin–Sphere model, indicating that both restriction and anisotropy are present in breast cancer tissues. There were no voxels in which ADC or DTI were the best models. Although variations in ADC often corresponded with variations in cellularity on histology, there were exceptions in which additional information was provided by the radius parameter *R* and intracellular volume fraction *f*_I_ from the Zeppelin–Sphere model. Regions of anisotropy corresponded to extracellular regions with aligned collagen on histology, but directions were only coherent over areas of approximately 1 mm and require high spatial resolution or diffusion techniques sensitive to sub‐voxel anisotropy^17^, ^57^ for their detection.

## Supporting information


**Supplementary Fig 1 A sample stacked histology volume shown in grayscale in three orthogonal views (shifted slides are evident as jagged black edges in the views along the bottom and right of the image). The registered diffusion‐weighted slice is shown overlaid in green and is tilted with respect to the slicing plane (bottom view), such that only a portion of the histology slice shown in the top left overlaps with the diffusion image.**
**Supplementary Fig 2 Fits (solid lines) to data (points) from a single voxel. In the left column, colours differentiate gradient separation times and shapes differentiate gradient durations; all orientations are plotted together. In the right column, the 42 directions of the two DTI scans are plotted, where cosψ describes the angle between the fitted primary diffusion direction (which may be different for different fits) and the gradient direction.**
**Supplementary Fig 3 Histograms of the posterior parameter distributions for each model obtained using the MCMC procedure with data for a single voxel with lower f**_**I**_ **than the voxel shown in Figure 3. The intracellular diffusion coefficient has a wider distribution than that of voxels with higher f**_**I**_ **, but the angular parameters (θ and ϕ) have narrower distributions, likely due to the larger extracellular signal contribution.**
**Supplementary Fig 4 Comparison of parameters for (top) Zeppelin‐Sphere and (middle row) Tensor‐Sphere showed similar values for most voxels. Correlation plots (bottom) demonstrated that most points where Zeppelin‐Sphere best explained the data (orange) had similar values in the two models, while points where Tensor‐Sphere best explained the data (cyan) deviated more from unity.**
**Supplementary Fig 5 Reproducibility data for two samples. (a) Registered intracellular volume fraction maps, f**_**I**_ **, from two separate scans of one sample (the voxels outside of the black outline were out‐of‐plane due to tilting of the slice). (b) A voxel‐by‐voxel correlation of f**_**I**_ **values shows that the best‐fit line (blue) deviates from unity (black), but has good correlation. (c) A boxplot of the distances of each voxel on the correlation plot from unity for all parameters (subplots) and models. Black shows the range for the grade 1 ductal/NST cancer sample shown in (a) and grey the values for the grade 3 mucinous carcinoma where low intracellular volume fraction gave poorly‐determined radius. Zeppelin‐X and Tensor‐X models had similar reproducibility for a given sample.**
**Supplementary Fig 6 Parametric maps for the ADC (2** ^**nd**^ **row) and selected parameters from the Zeppelin‐Sphere model (rows 3‐5) for the three predominantly fatty samples. Scale bar represents 5 mm.**
**Supplementary Fig 7 H&E stained histology alongside colour FA maps from the zeppelin portion of Zeppelin‐Sphere fit for the three predominantly fatty samples**

Supporting info item

## APPENDIX 1

### 1.1

Mathematical descriptions of the model components are presented here for the pulsed gradient spin echo (PGSE) sequence. Compartments are assumed to have slow exchange of water between them, such that the total signal in a voxel *S* is the sum of all signal compartments *S*_*i*_ weighted by their respective fractions *f*_*i*_: $S=S_{0}e^{-\frac{\mathrm{TE}}{T2}}\sum_{i}f_{i}S_{i}$, where *S*_0_ is the equilibrium signal intensity, TE is the echo time and T2 is the *T*_2_ relaxation time constant (*S*_0_ and T2 are fitted parameters in all models).

The Ball compartment is equivalent to the signal for Gaussian diffusion: $S_{\text{Ball}}=e^{{-\mathrm{bD}}_{1}}$ with *b* = (*γgδ*)^2^(*Δ* − *δ*/3), where *γ* is the gyromagnetic ratio, *g* is the gradient strength, *δ* is the gradient duration and *Δ* is the gradient separation.

The Zeppelin is the product of signal along the primary diffusion direction and the direction perpendicular to this: $S_{\text{Zeppelin}}=e^{{-b\;{\text{cos}}^{2}\psi D}_{1}}\;e^{{-b\;\left(1-{\text{cos}}^{2}\psi \right)D}_{2}}$, where *ψ* is the angle between the normalised gradient direction $\hat{g}$ and the parallel diffusion direction $\hat{n}$ defined in spherical co‐ordinates with *θ* and *ϕ*: $\cos\psi =\hat{g}\cdot \hat{n}$, $\hat{n}=\left(\sin\theta \cos\phi ,\sin\theta \sin\phi ,\cos\theta \right)$.

The Tensor signal is given by $S_{\text{Tensor}}=e^{{-b\;{\text{cos}}^{2}\psi_{1}D}_{1}}\;e^{{-b\;{\text{cos}}^{2}\psi_{2}D}_{2}}\;e^{{-b\;{\text{cos}}^{2}\psi_{3}D}_{3}}$, where cos*ψ*_*i*_ is the angle between the gradient direction and the *i*th eigenvector from the diagonalisation of the diffusion matrix, such that ${\hat{n}}_{1}=\left(\sin\theta \cos\phi ,\sin\theta \sin\phi ,\cos\theta \right)$, ${\hat{n}}_{2}=\hat{k}\cos\alpha +\left(\hat{k},\times ,{\hat{n}}_{1}\right)\sin\alpha +{\hat{n}}_{1}\left(\hat{k},▪,{\hat{n}}_{1}\right)\left(1,-,\cos\alpha \right)$, $\hat{k}=\left(\sin\theta +\frac{\pi }{2}\cos\phi ,\sin\theta +\frac{\pi }{2}\sin\phi ,\cos\theta +\frac{\pi }{2}\right)$ is a vector orthogonal to ${\hat{n}}_{1}$ rotated by an angle *α* in that plane and therefore ${\hat{n}}_{3}\equiv {\hat{n}}_{1}\times {\hat{n}}_{2}$.

The Sphere signal is calculated using the Gaussian phase distribution approximation:^59^ $S_{\text{Sphere}}=\exp\left(-2\gamma^{2}g^{2}\sum_{m}\frac{2{\beta_{m}}^{2}D_{1}\delta -2+2Y\left(\delta \right)+2Y\left(\Delta \right)-Y\left(\Delta -\delta \right)-Y\left(\Delta +\delta \right)}{{D_{1}}^{2}{\beta_{m}}^{6}\left({\beta_{m}}^{2}R^{2}-2\right)}\right)$. Here, $Y\left(x\right)=e^{-{\beta_{m}}^{2}D_{1}x}$, *β*_*m*_ is the *m*th root of $J_{\frac{3}{2}}\left(\beta_{m},R\right)-\beta_{m}RJ_{\frac{5}{2}}\left(\beta_{m},R\right)=0$ and *J*_*ν*_ is the Bessel function of the first kind, order *ν*. The summation was carried out over the first 31 roots of the equation.
