# Supplementary material for: Microstructural models for diffusion MRI in breast cancer and surrounding stroma: an ex vivo study
Source: NMR Biomed. 2016 Dec 21;30(2):e3679. doi: 10.1002/nbm.3679 (PMC5244665; doi:10.1002/nbm.3679)
Supplement: Supplementary file 1 — Supporting info item [file NBM-30-0-s001.doc]

# Supplementary Information


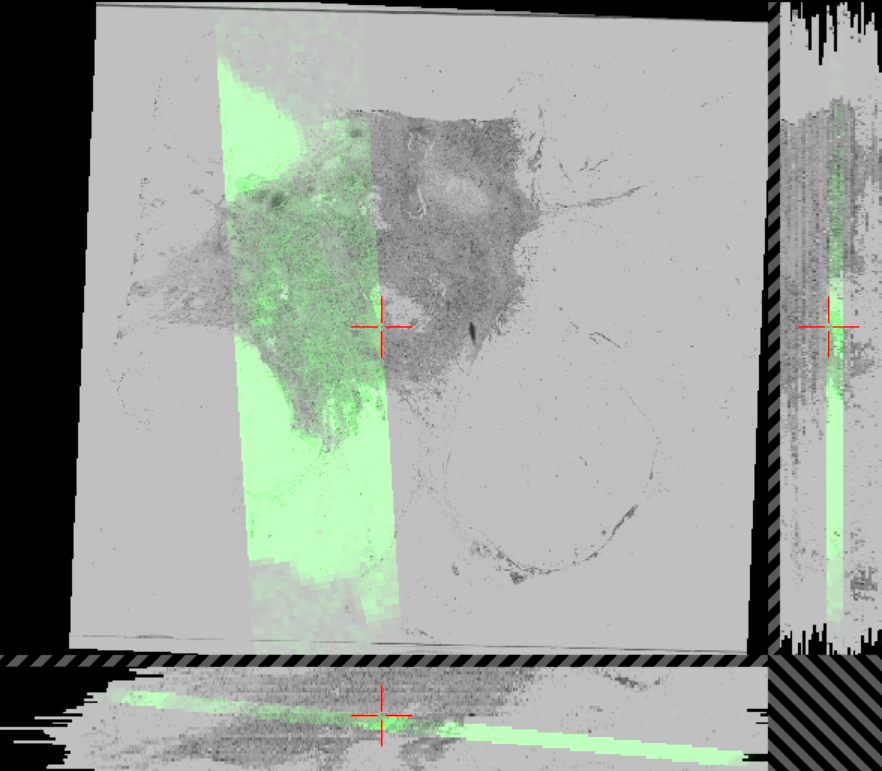


Supplementary Fig 1 A sample stacked histology volume shown in grayscale in three orthogonal views (shifted slides are evident as jagged black edges in the views along the bottom and right of the image). The registered diffusion-weighted slice is shown overlaid in green and is tilted with respect to the slicing plane (bottom view), such that only a portion of the histology slice shown in the top left overlaps with the diffusion image.


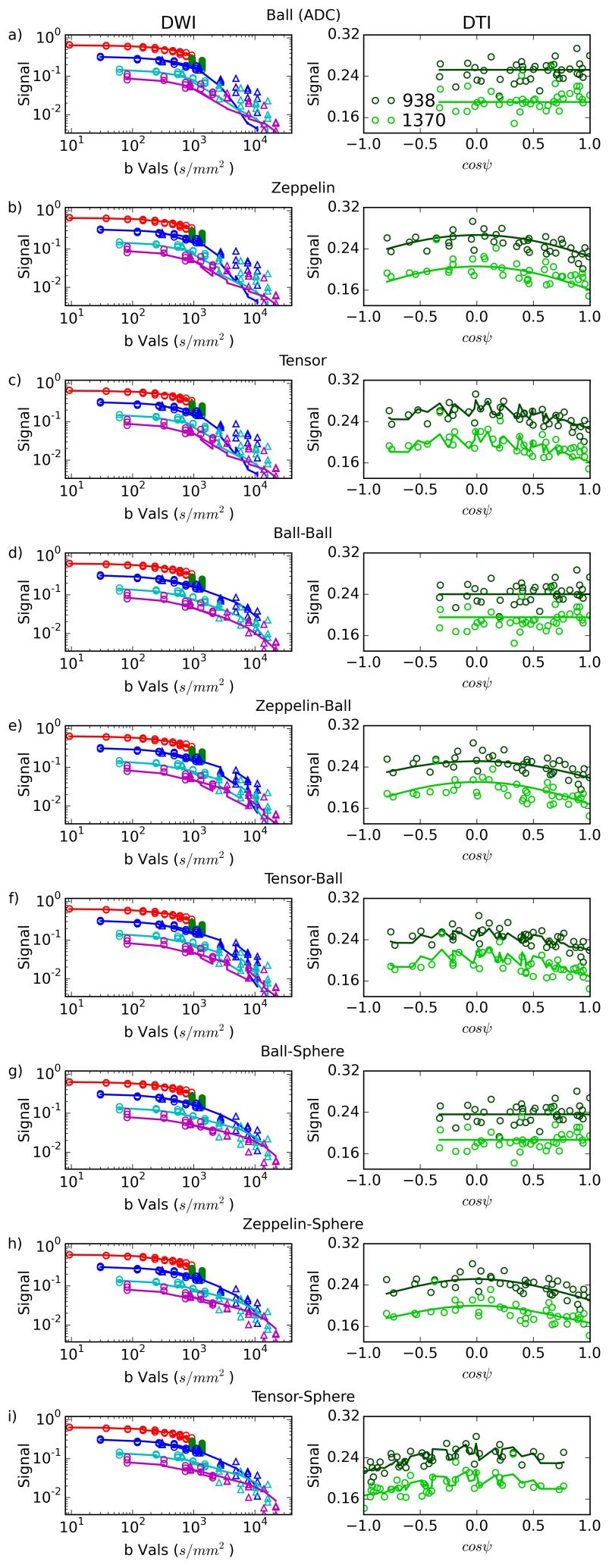


Supplementary Fig 2 Fits (solid lines) to data (points) from a single voxel. In the left column, colours differentiate gradient separation times and shapes differentiate gradient durations; all orientations are plotted together. In the right column, the 42 directions of the two DTI scans are plotted, where cosψ describes the angle between the fitted primary diffusion direction (which may be different for different fits) and the gradient direction.


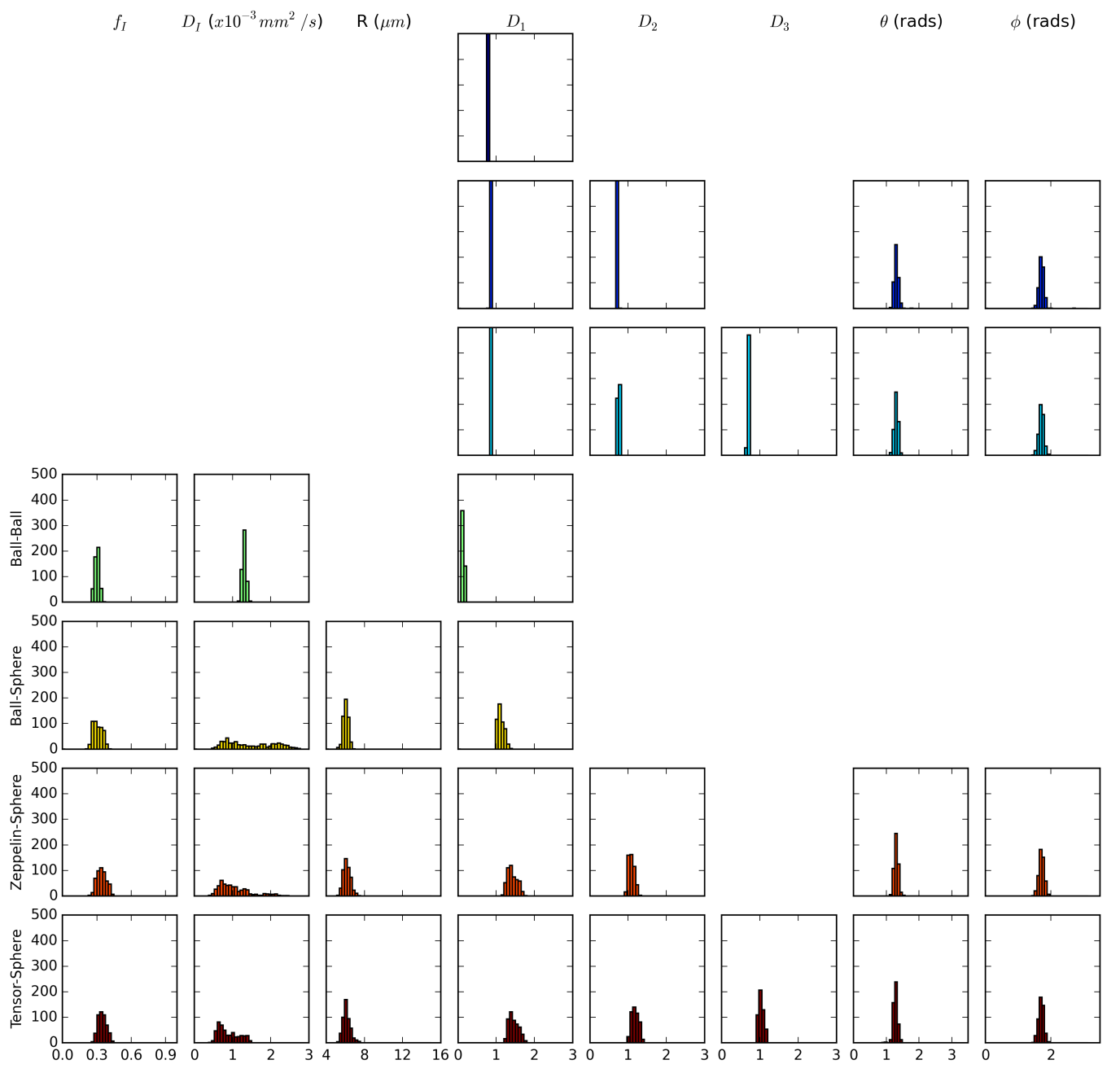


Supplementary Fig 3 Histograms of the posterior parameter distributions for each model obtained using the MCMC procedure with data for a single voxel with lower f_I_ than the voxel shown in Figure 3. The intracellular diffusion coefficient has a wider distribution than that of voxels with higher f_I_, but the angular parameters (θ and φ) have narrower distributions, likely due to the larger extracellular signal contribution.


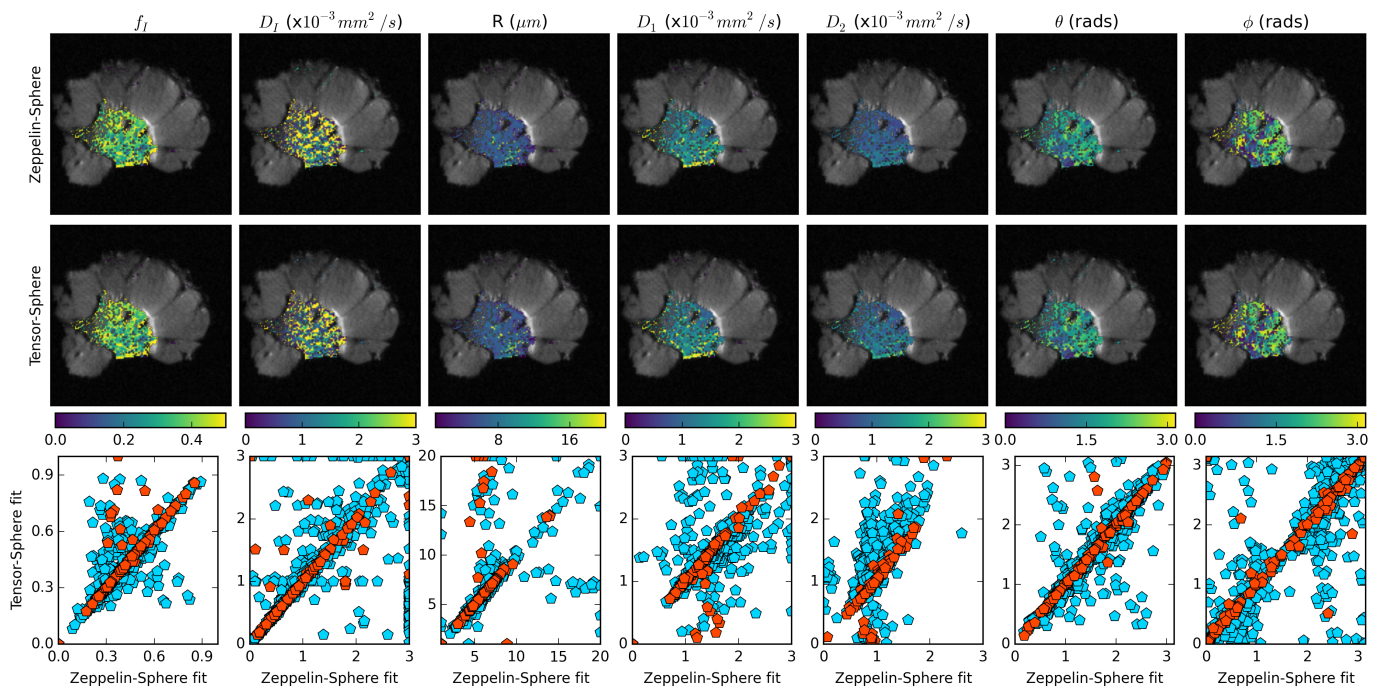


Supplementary Fig 4 Comparison of parameters for (top) Zeppelin-Sphere and (middle row) Tensor-Sphere showed similar values for most voxels. Correlation plots (bottom) demonstrated that most points where Zeppelin-Sphere best explained the data (orange) had similar values in the two models, while points where Tensor-Sphere best explained the data (cyan) deviated more from unity.


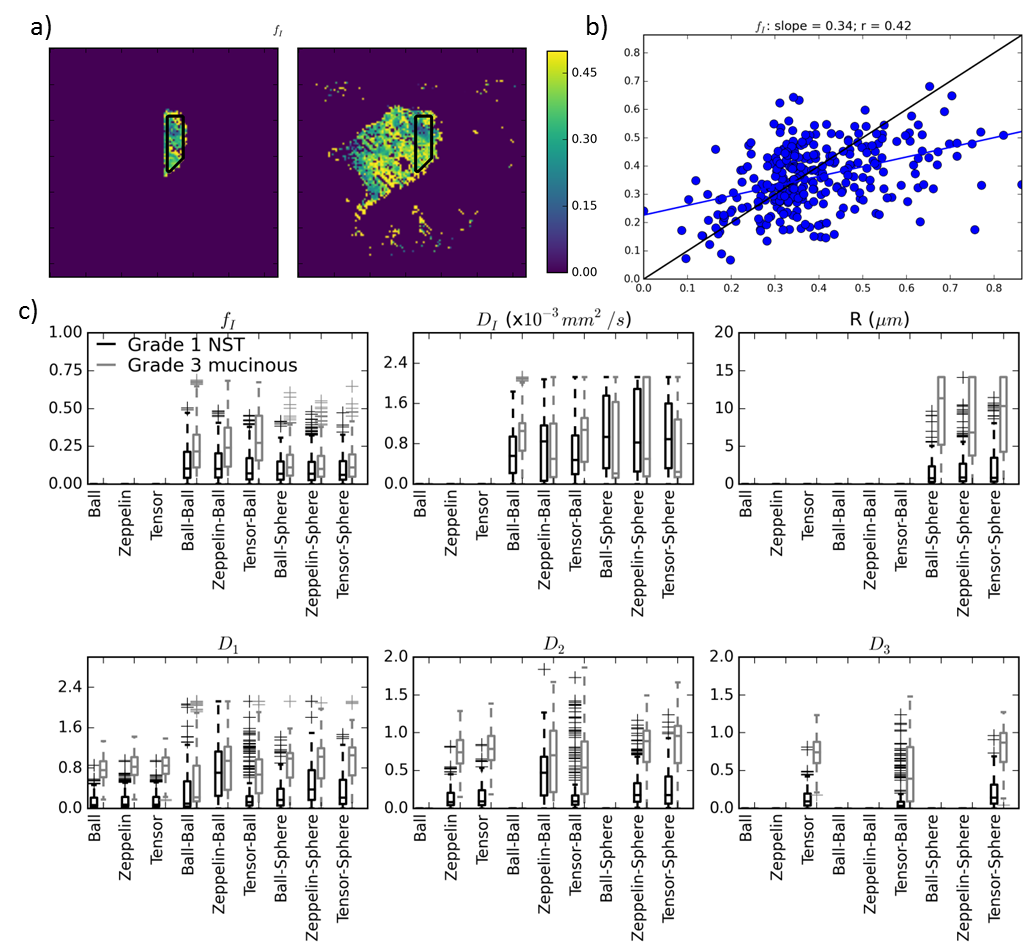


Supplementary Fig 5 Reproducibility data for two samples. (a) Registered intracellular volume fraction maps, f_I_, from two separate scans of one sample (the voxels outside of the black outline were out-of-plane due to tilting of the slice). (b) A voxel-by-voxel correlation of f_I_ values shows that the best-fit line (blue) deviates from unity (black), but has good correlation. (c) A boxplot of the distances of each voxel on the correlation plot from unity for all parameters (subplots) and models. Black shows the range for the grade 1 ductal/NST cancer sample shown in (a) and grey the values for the grade 3 mucinous carcinoma where low intracellular volume fraction gave poorly-determined radius. Zeppelin-X and Tensor-X models had similar reproducibility for a given sample.


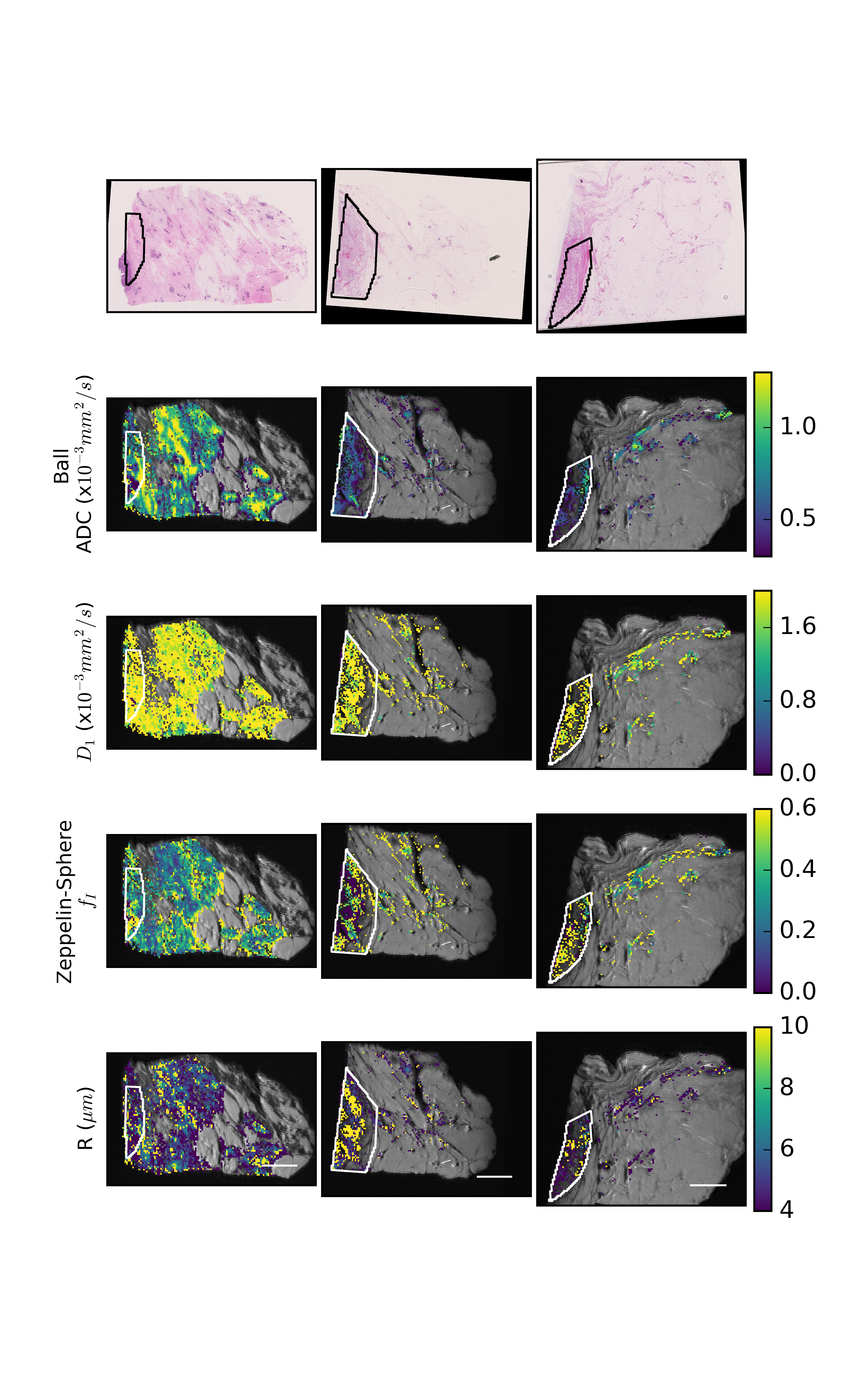


Supplementary Fig 6 Parametric maps for the ADC (2^nd^ row) and selected parameters from the Zeppelin-Sphere model (rows 3-5) for the three predominantly fatty samples. Scale bar represents 5 mm.


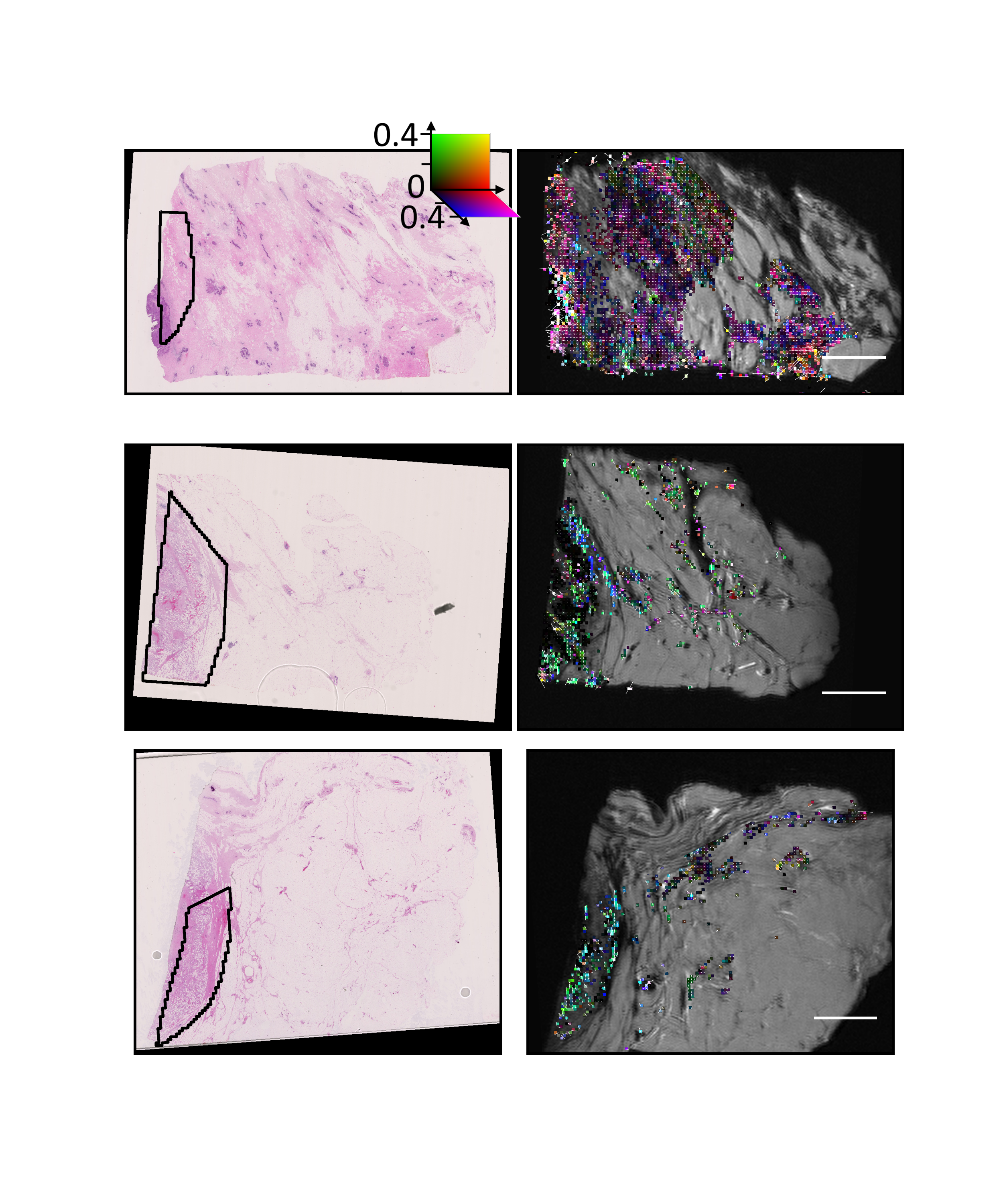


Supplementary Fig 7 H&E stained histology alongside colour FA maps from the zeppelin portion of Zeppelin-Sphere fit for the three predominantly fatty samples
